# Efficacy and safety of Hou Gu Mi Xi in patients with spleen qi deficiency syndrome who underwent radical gastrectomy for gastric cancer: protocol for a multicenter, randomized, double-blind, placebo-controlled trial

**DOI:** 10.1186/s13063-019-3429-x

**Published:** 2019-06-10

**Authors:** Xu Zhou, Dong-mei Yan, Wei-feng Zhu, Wen-jun Liu, He-yun Nie, Sheng Xu, Yi-ping Jiang, Kun-he Zhang, Ying Fu, Yi-ye Wan, Xin-yu Yu, Hong Li, Xin Sun, Xiao-fan Chen

**Affiliations:** 1 Evidence-based Medicine Research Center, School of Basic Medical Sciences, Jiangxi University of Traditional Chinese Medicine, Mei Ling Da Dao No. 1688, Nanchang, 330004 Jiangxi China; 2 School of Food Science and Engineering, Inner Mongolia Agricultural University, Inner Mongolia, China; 3 Department of Spleen, Stomach, Liver and Gallbladder Diseases, Affiliated Hospital of Jiangxi University of Traditional Chinese Medicine, Jiangxi, China; 4 Department of Gastroenterology, First Affiliated Hospital of Nanchang University, Jiangxi, China; 5 Department of Traditional Chinese Medicine, Second Affiliated Hospital of Nanchang University, Jiangxi, China; 6 Third Department of Oncology, Jiangxi Provincial Cancer Hospital, Jiangxi, China; 7 Chinese Evidence-based Medicine Center, West China Hospital, Sichuan University, Sichuan, China

**Keywords:** Hou Gu Mi Xi, Randomized controlled trial, Traditional Chinese medicine, Gastric cancer, Radical gastrectomy, Spleen qi deficiency

## Abstract

**Background:**

Spleen qi deficiency (SQD), a syndrome based on traditional Chinese medicine (TCM) theory, is common in patients after radical gastrectomy. SQD manifests with chronic gastrointestinal disorders and systemic symptoms and is challenging to manage. Hou Gu Mi Xi (HGMX) is a dietary TCM formula for SQD. This study aims to evaluate the efficacy and safety of HGMX in patients with SQD who have undergone radical gastrectomy for gastric cancer.

**Methods and design:**

This study is a multicenter, randomized, double-blind, placebo-controlled trial. One hundred thirty patients with SQD who have undergone radical gastrectomy for gastric cancer will be assigned to receive either HGMX or placebo for 2 years. The main outcome will be changes in SQD symptoms assessed by the Spleen Qi Deficiency Symptoms Grading and Quantifying Scale. The secondary outcomes will be changes in quality of life assessed by the Short Form 36 scale, performance status as assessed by the Eastern Cooperative Oncology Group Performance Status scale, body weight, and body mass index. Progression-free survival will also be assessed as a secondary outcome. Adverse events (AEs), severe AEs, and study withdrawal due to AEs will be recorded to evaluate the safety of HGMX.

**Discussion:**

The results of this trial will provide initial evidence for the use of HGMX as an alternative and complementary intervention to manage chronic postoperative complications in patients who have undergone radical gastrectomy for gastric cancer.

**Trial registration:**

ClinicalTrials.gov, NCT03025152. Registered on 17 January 2017.

**Electronic supplementary material:**

The online version of this article (10.1186/s13063-019-3429-x) contains supplementary material, which is available to authorized users.

## Background

Although the incidence of gastric cancer is decreasing annually because of the popularization of *Helicobacter pylori* detection and eradication and improvements in living conditions, it represents one of the largest disease burdens worldwide [1]. According to the statistics reported by the World Health Organization in 2016, gastric cancer accounts for 1.53% of deaths worldwide, second only to lung cancer [2]. Due to the high *H. pylori* infection rate, dietary habits, and susceptibility genes, approximately 50% of gastric cancer cases worldwide occur in East Asian populations [3].

Radical gastrectomy, the standard treatment with a curative intent for patients with early-stage gastric cancer, can substantially prolong disease-free and overall survival [4]. However, chronic complications after the surgery and adjuvant chemoradiotherapy, such as chronic gastrointestinal disorders (e.g., abdominal distension and diarrhea) and general symptoms (e.g., physical fatigue and mental issue), are common and may lead to discomfort, malnutrition, hypoimmunity, decreased quality of life, and even cancer relapse [5]. Although the management of chronic complications is crucial for improving the prognosis of patients with gastric cancer, there are no specific interventions in the current guidelines for treating the complications except for surveillance and nutritional support [4, 6, 7]. Therefore, alternative and complementary therapies are popular in clinical practice [8, 9]; in particular, traditional Chinese medicine (TCM) is widely used to treat postgastrectomy complications in China, which has the heaviest disease burden of gastric cancer globally [10–12].

In TCM theory, the spleen (also written as “pi”, an abstract digestive organ in TCM that differs from the Western medical definition of the spleen as an immune organ) is responsible for transporting food and water, and qi is the vital life force produced by vital activity [13]. Patients who have undergone radical gastrectomy and chemoradiotherapy can easily suffer damage to the spleen and qi, leading to a spleen qi deficiency (SQD) syndrome, the typical symptoms of which include stomach and abdominal distension, dyspepsia, chest and stomach distress, borborygmus, diarrhea, fatigue, weakness, and weight loss [11, 14].

Dietary TCM therapy is a healthcare approach that penetrates Chinese people’s daily life. For example, people like eating medlar to tonify the kidney and liver and avoid contraindicated stimulating food (e.g., mutton, cock, and carp) to prevent the progression of inflammation or cancer. However, the efficacy of these dietary therapies is limited by their simple structure. Theoretically, dietary TCM formulas composed of multiple herbs will have a better efficacy than the simple forms.

Shen Ling Bai Zhu San, a classic TCM formula with the effects of invigorating the spleen and replenishing qi, has been validated to be effective and safe in treating SQD syndrome by empirical evidence for more than 3000 years [15]. It consists of *Renshen* (ginseng), *Fuling* (tuckahoe), *Baizhu* (atractylodes), *Yiyiren* (coixenolide), *Shanyao* (Chinese yam), *Lianzi* (lotus seed), *Sharen* (amomum), *Jiegen* (Platycodon), *Baibiandou* (white hyacinth bean), and *Gancao* (licorice), all of which are herbs that can be eaten as food (i.e., dietary herbs) except for *Baizhu*. Therefore*,* Shen Ling Bai Zhu San is appropriate for modification as a dietary TCM formula.

There is some evidence supporting the gastrointestinal protection and regulatory effects of Shen Ling Bai Zhu San. Animal studies have suggested that Shen Ling Bai Zhu San has the effects of regulating gut flora and increasing gastrointestinal motility [16]. A population-based survey from Taiwan showed that the number of Shen Ling Bai Zhu San prescriptions ranked fourth among all TCM formulas among 46,119 patients with gastric cancer and significantly decreased mortality in patients undergoing both total and partial gastrectomy during a 3.56-year median follow-up period [17]. A guideline developed by the Beijing Academy of Traditional Chinese Medicine recommends using Shen Ling Bai Zhu San as an adjuvant therapy for treating gastric cancer based on randomized controlled trial (RCT) evidence [18]. However, all RCTs supporting the efficacy of Shen Ling Bai Zhu San in the guidelines are of low quality.

A currently used dietary TCM formula called Hou Gu Mi Xi (HGMX) was developed by Jiangzhong Pharmaceutical (group) Co., Ltd. (Jiangxi, China), and is modified from Shen Ling Bai Zhu San. To modify the dietary formulation, HGMX does not contain *Baizhu*, which cannot be taken as a dietary supplement, and instead contains an alternative herb (*Jupi*) to maintain the main effects. As a result, all the herbs in HGMX can be used as both a drug and a dietary supplement according to the regulations in China. HGMX also increases the proportion of *Fuling*, *Yiyiren*, and *Baibiandou* to improve the effects of tonifying the spleen and dispelling dampness and decreases the proportion of *Renshen*, *Shanyao*, *Lianzi*, and *Gancao* because these herbs can cause dampness, which may affect the efficacy. The comparative components of HGMX and Shen Ling Bai Zhu San are presented in Table 1. In HGMX, *Renshen and Fuling* are the principal drugs, with *Renshen* having the main effects of tonifying qi and reinforcing the spleen and *Fuling* being responsible for dispelling dampness. *Yiyiren, Shanyao*, and *Lianzi* are used as adjuvant drugs to enhance the effects of the principal drugs, and *Sharen, Jiegen, Baibiandou, Gancao,* and *Jupi* help with the regulation and utilization of the entire formula. In addition, long-term patient compliance with Shen Ling Bai Zhu San is limited by the powder preparations and poor taste. To improve patient compliance, the HGMX preparation is changed from a powder to oatmeal by adding japonica rice and oats so that it can be brewed as a rice paste and conveniently eaten for breakfast. Japonica rice and oats also have slight effects of tonifying the spleen, which may enhance the efficacy of the herbs in HGMX.

**Table 1:** Detailed components of Hou Gu Mi Xi and Shen Ling Bai Zhu San

Hou Gu Mi Xi			Shen Ling Bai Zhu San
Component	Part used	Proportion	Proportion
*Renshen* (ginseng)	Root	5.0%	12.9%
*Fuling* (tuckahoe)	Sclerotium	20.0%	12.9%
*Yiyiren* (coixenolide)	Seed	20.0%	6.5%
*Shanyao* (Chinese yam)	Root	10.0%	12.9%
*Lianzi* (lotus seed)	Fruit	10.0%	6.5%
*Sharen* (amomum)	Fruit	1.0%	6.5%
*Jiegen* (Platycodon)	Stem	5.0%	6.5%
*Baibiandou* (white hyacinth bean)	Seed	20.0%	9.7%
*Gancao* (licorice)	Stem	5.0%	12.9%
*Jupi* (orange peel)	Pericarp	5.0%	*Baizhu* (atractylodes) 12.9%

Every 30 g of Hou Gu Mi Xi contains an additional 13 g of early rice and 6.9 g of oats, in addition to 10.1 g of herbs

Based on TCM theory, we hypothesize that HGMX will be beneficial and safe for SQD syndrome caused by radical gastrectomy with better long-term patient compliance. However, because of the modifications, the RCT evidence for Shen Ling Bai Zhu San cannot be directly applied to HGMX, and furthermore, this evidence is not reliable. To evaluate the efficacy and safety of HGMX in patients with SQD and gastric cancer who have undergone radical gastrectomy, we plan to perform a 2-year randomized placebo-controlled trial (RCT). The results of this RCT will provide evidence for HGMX as an alternative and complementary therapy for the long-term management of chronic postgastrectomy complications.

## Methods/design

### Study design and recruitment

**Fig. 1:**
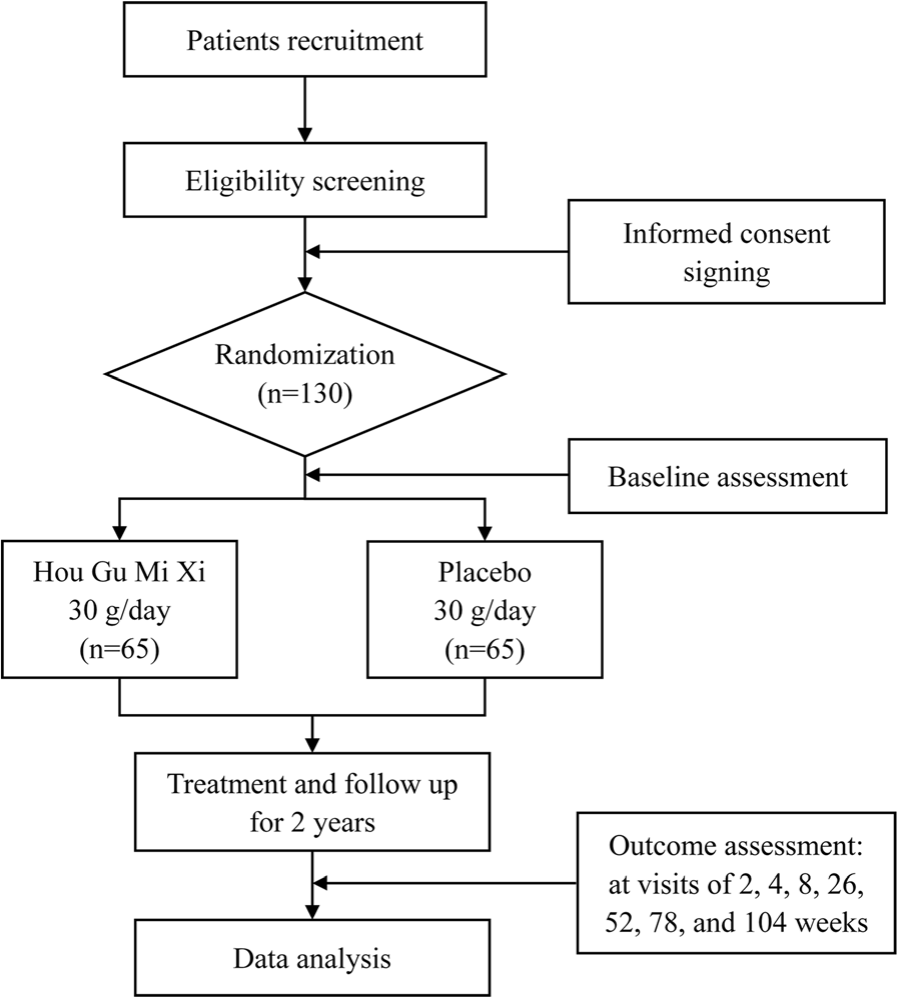
Flowchart of the study

This study is a multicenter, randomized, double-blind, parallel, placebo-controlled trial. The flowchart of the study is presented in Fig. 1. The reporting of the protocol follows the Standard Protocol Items: Recommendations for Interventional Trials (SPIRIT) guidelines [19]. Additional file 2 contains the SPIRIT checklist. Four tertiary hospitals have agreed to participate in the trial, namely, the First Affiliated Hospital of Nanchang University, the Second Affiliated Hospital of Nanchang University, Jiangxi Hospital of Traditional Chinese Medicine, and Jiangxi Provincial Cancer Hospital.

The trial will recruit participants through Internet advertisements and posters in the hospitals. Patients who have undergone radical gastrectomy for gastric cancer and show interest in participating in the trial will be evaluated by an attending clinician to determine their eligibility. The clinician will inform the patient of the detailed study objectives, the study process, and the potential benefits and risks. All participants will sign an informed consent form prior to participation.

### Patient eligibility

An eligible patient should meet all of the following criteria:

- Age between 18 and 70 years
- Pathological diagnosis of gastric cancer
- Underwent radical gastrectomy, radiotherapy, chemotherapy, and treatment of surgical complications (such as leaks, strictures, and marginal ulcers) 2 weeks to 1 year previously
- Fair performance status indicated by a score on the Eastern Cooperative Oncology Group (ECOG) questionnaire ≤1.
- Signed the informed consent form
- Diagnosed with SQD syndrome based on TCM theory as follows:
  - Primary symptoms of spleen deficiency: (1) poor appetite, (2) stomach or abdominal distention, and (3) abnormal stools (loose stools or diarrhea)
  - Primary symptoms of qi deficiency: (1) physical fatigue and weakness and (2) mental fatigue and taciturnity
  - Secondary symptoms: (1) loss of taste or hypodipsia, (2) stomach or abdominal pain, (3) stomach tightness, (4) nausea or vomiting, (5) abnormal bowel sounds, (6) leanness or puffiness, (7) sallow complexion, (8) powerless defecation, and (9) facial or limb edema
  - Auxiliary symptoms: pale, swollen, or scalloped tongue with a thin and white coating.
  Patients who meet two primary symptoms of spleen deficiency plus two primary symptoms of qi deficiency, two primary symptoms of spleen deficiency plus one primary symptom of qi deficiency plus one auxiliary symptom, or one primary symptom of spleen deficiency plus one primary symptom of qi deficiency plus two secondary symptoms plus one auxiliary symptom will be diagnosed with SQD syndrome.

An eligible patient should not meet any of the following exclusion criteria:

- Stage IV gastric cancer according to the Japanese classification criteria [20]
- Completed radical gastrectomy, radiotherapy, and chemotherapy more than 1 year previously
- Impaired liver function (total bilirubin > 2 × upper limit of normal (ULN), alanine transaminase > 2 × ULN, or aspartate aminotransferase > 2 × ULN), kidney function (serum creatinine > 2 × ULN), or hematopoiesis (neutrophil count < 0.5 × 10^9^/L, thrombocyte count < 20 × 10^9^/L, or absolute reticulocyte count < 15 × 10^9^/L)
- Obviously abnormal electrocardiogram
- Severe mental disorder
- Other severe diseases (e.g., multiple organ failure or acquired immune deficiency syndrome)
- Pregnant or breastfeeding
- Allergy to the test sample
- Unwilling to provide personal information.

### Randomization and blinding

Eligible patients will be randomly assigned at a 1:1 ratio to receive either HGMX or placebo. We generated the random sequence using computer software. An independent randomization management center is responsible for sequentially assigning the random number and distributing trial samples to patients to ensure that the process of allocation is concealed. Clinicians, patients, data collectors, and outcome assessors will be blinded to the group assignment. The allocation will be unblinded if a severe adverse event occurs and when the final data analysis is complete.

### Interventions and concomitant treatments

Eligible patients will receive either 30 g/day HGMX or 30 g/day placebo taken orally for 2 years. Every 30 g of HGMX contains 10.1 g of herbal materials. The placebo consists of early rice and oats and is identical to HGMX in terms of preparation, packaging, labeling, and color but is slightly different in taste and smell; according to our simulation test, the two treatments should be impossible to distinguish. The test samples will be distributed to patients once per month. Other Chinese herbal medicines and Western drugs to treat gastrointestinal symptoms (e.g., proton pump inhibitors, prokinetics) are prohibited. The patients will be asked to sign an integrity commitment; they should truthfully report whether they used a treatment outside the regimen at any time. This information will be recorded and used for sensitivity analyses.

Both groups will undergo surveillance of gastric cancer according to the Japanese Gastric Cancer Treatment Guidelines 2014 [4] and continuous concomitant care for other comorbidities (such as hypertension, diabetes mellitus, and osteoarthritis) during the study follow-up period. Concomitant treatments will be recorded at each visit.

### Outcome measurements

#### Primary outcome

The primary outcome is the change in the total score of the Spleen Qi Deficiency Symptoms Grading and Quantifying scale (SQD scale). The SQD scale is a modified version of the classic scale for assessing the degree of SQD in the Clinical Guideline of New Drugs for Traditional Chinese Medicine [21]. The SQD scale consists of 17 items regarding symptoms of SQD: (1) stomach distension, (2) abdominal distension, (3) physical fatigue and weakness, (4) mental fatigue and taciturnity, (5) loss of appetite, (6) abnormal stools, (7) stomach pain, (8) stomach tightness, (9) abdominal pain, (10) acid reflux, (11) belching, (12) nausea and vomiting, (13) abnormal bowel sounds, (14) powerless defecation, (15) sallow complexion, (16) loss of taste and hypodipsia, and (17) facial and limb edema. Items 1–6 are the main symptoms; the other items are secondary symptoms. The items are assessed for duration (0–3 points, only available for items 1–2 and 7–9), severity (0–3 points, not available for item 13), frequency per day (0–4 points; 0–3 points for item 5), and frequency per week (0–5 points). The total score is the sum of the scores of each item (the main symptoms are scored twice) and ranges from 0 to 292. A higher score indicates a more severe symptom. Compared with the classic scale, we added four items (items 1, 7, 10, and 11), defined the main symptoms according to TCM theory, and assessed each item for duration, severity, and frequency per day and per week rather than only for severity. The details of the SQD scale are presented in Additional file 1.

#### Secondary outcomes

The secondary outcomes are:

- Quality of life, assessed by a change in the Short Form 36 scale score [22]
- Performance status, assessed by a change in the ECOG questionnaire score [23]
- Changes from baseline in body weight and body mass index
- Progression-free survival, assessed by pathological examination, computed tomography, and/or magnetic resonance imaging.

#### Safety outcomes

The safety outcomes are as follows:

- Adverse events (AEs), such as abnormal results (greater or less than 2 × normal reference interval) in routine blood, urine, and stool tests; liver function tests (alanine transaminase, aspartate aminotransferase, total bilirubin, direct bilirubin, and indirect bilirubin); kidney function tests (serum creatinine and urea nitrogen); coagulation function (prothrombin time, activated partial thromboplastin time, thrombin time, and fibrinogen); or electrocardiograms; as well as any other new-onset symptoms or diseases related or unrelated to the intervention
- Severe AEs (SAEs) are those AEs that lead to new or prolonged hospitalization, disability, admission to the intensive care unit, mortal danger, or death. SAEs will be reported to the principal investigator, the ethics committee of the hospital, and the Jiangxi Food and Drug Administration within 24 h
- Withdrawal due to AEs.

### Other assessments

Demographic profiles and histories of patients will be recorded at the baseline visit, including sex, age, marital status, education level, smoking and drinking habits, comorbidities, pathological type of gastric cancer, gastric cancer stage, type of resection surgery, chemoradiotherapy regimen, and medication history for the past 3 months.

### Study visits and outcome assessments

**Fig. 2:**
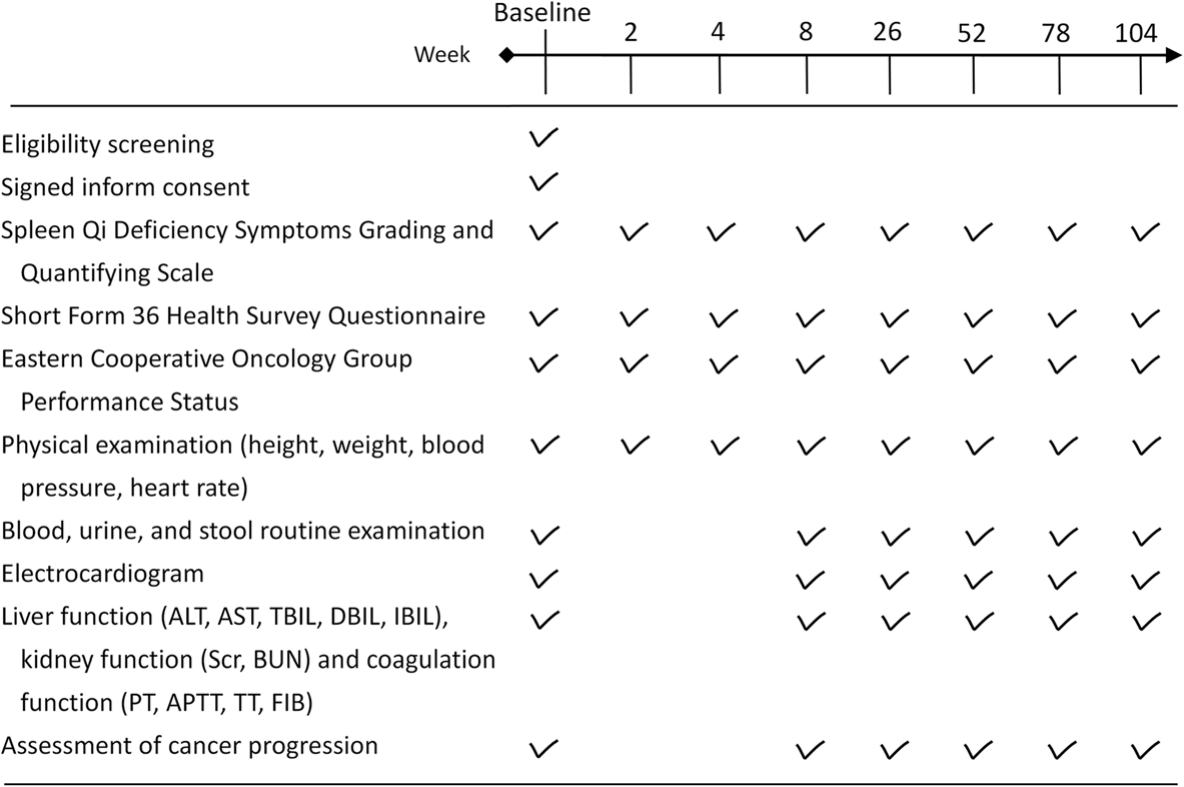
Schedule of the trial process. Abbreviations: *ALT* alanine transaminase, *AST* aspartate aminotransferase, *TBIL* total bilirubin, *DBIL* direct bilirubin, *IBIL* indirect bilirubin, *SCr* serum creatinine, *BUN* blood urea nitrogen, *PT* prothrombin time, *APTT* activated partial thromboplastin time, *TT* thrombin time, *FIB* fibrinogen

The duration of follow-up will be 2 years. Patients will visit at baseline and at 2, 4, 8, 26, 52, 78, and 104 weeks. At each visit, questionnaires and physical examinations will be completed, self-reported AEs will be recorded, and compliance will be assessed. At 8, 26, 52, 78, and 104 weeks, laboratory examinations and electrocardiograms will be performed, and cancer progression will be assessed. A detailed schedule of the trial is presented in Fig. 2.

### Sample size estimation

This RCT is a superiority trial, and an appropriate formula was used to estimate sample size [24]. Based on the results of our preliminary observational study and expert advice, we assume an α of 0.05, a 1 –β of 0.80, a between-group difference in primary outcome (total SQD scale score) δ of 8.0, a pooled standard deviation of 14.5, and an allowable dropout rate of 20%. The total sample size is expected to be 130 (65 in each group).

## Statistical analysis

Data analysis will be performed with Stata v14.0 (StataCorp LLC, College Station, TX, USA). The main primary outcome analysis will be based on the intention-to-treat data set, where the missing values will not be imputed and the patients who took unscheduled medications will be kept in their original group. We will also perform four sets of sensitivity analyses to test the robustness of the results of the main analyses, including (1) using the per protocol data set; (2) imputing missing data using various methods dependent on the proportion and distribution of the missing data; (3) excluding patients who become aware of the grouping accidentally during the trial, which also aims to estimate the placebo effects; and (4) excluding patients who took unscheduled medications. The analysis of secondary outcomes will be based on the per protocol data set, and the analysis of safety outcomes will be based on the safety data set only, both with no imputation. Interim data analyses will be performed after weeks 8 and 52.

Descriptive analysis will be conducted to explore the means and standard deviations of the continuous variables. We will assess the treatment effects by comparing the mean between-group differences of the continuous endpoints with a normal distribution using the independent *t* test. The skewed continuous variables will be described using medians and interquartile ranges. We will use the Mann-Whitney *U* test to assess the equivalency of the skewed continuous variable distributions. Moreover, we will describe categorical variables through their frequencies and percentages. We will evaluate the between-group differences using Pearson’s chi-square test for the cases with expected counts greater than 5 in more than 20% of cells and greater than 1 in any cell or Fisher’s exact test otherwise. We will perform subgroup analyses stratified by gastric cancer stage and chemoradiotherapy regimen for the primary outcome. We set α = 0.05 as the nominal type I error rate, which will be adjusted for the multiple *t* test using the O’Brien-Fleming group sequential method [25].

### Data collection and monitoring

All investigators involved in the trial will receive research training based on standard operating procedures. An independent data monitoring committee (DMC) at Jiangxi University of Traditional Chinese Medicine will be founded and be responsible for supervising the entire trial process. Data collection, such as questionnaire surveys, physical examinations, biological samples, and laboratory examinations, will be performed by qualified physicians at each research center. Student volunteers, as clinical research coordinators, from Jiangxi University of Traditional Chinese Medicine will be in charge of contacting patients for timely follow-up. Each research hospital will have a clinical research associate from the Jiangxi University of Traditional Chinese Medicine who will be in charge of monitoring patient compliance and case report form completeness and will address issues during the trial. The original case report forms will be inputted into the online database by two clinical research coordinators in duplicate. Personal information for all patients will be anonymized. The database will be password protected and will be managed by the DMC. An independent analyst will have access to the database only during the data analysis period. The DMC will also have the authority to discontinue the entire trial according to the following standards: (1) HGMX does not show any efficacy toward SQD symptoms up to 8 weeks of follow-up, as indicated by an absolute difference < 5 on the SQD scale score between the two groups (this difference is estimated based on the unpublished results of our other trial assessing HGMX for nonorganic gastrointestinal disorders); (2) HGMX does not show statistically significant efficacy for any outcome up to 52 weeks of follow-up compared with placebo; and (3) any severe AE related to HGMX occurs.

### Compliance and withdrawal

To improve patient compliance, we will offer all test samples (HGMX and placebo) and scheduled examinations free of charge, and patients will receive a travel allowance of 100 yuan at each visit. At the recruitment stage, researchers will emphasize the length of the 2-year treatment and follow-up to patients who are willing to join the trial. The patients will be allowed to complete each follow-up within ±3 days of the scheduled date for the weeks 2, 4, and 8 visits and within ±7 days for the weeks 26, 52, 78, and 104 visits. The used packaging bags will be reclaimed to check patients’ compliance.

Patients will be withdrawn from the trial for the following reasons:

- Cancer progression and restarting of radiotherapy or chemotherapy
- Recurrence of surgical complications
- Continuous deterioration of SQD symptoms
- Poor compliance, indicated by the use of less than 80% of the prescribed trial drug or more than two missed visits
- Active withdrawal. Patients can actively withdraw from the trial at any time for any reason, and subsequent treatment will not be affected

## Discussion

Some previous RCTs assessed the effects of Shen Ling Bai Zhu San, the basic formula of HGMX, for treating patients with gastric cancer. These RCTs showed promising results, such as reduction in the incidence and severity of postoperative ileus, irritable bowel syndrome, and anemia [26], remission of gastrointestinal symptoms [27], and improvement in quality of life [28], which could also support the efficacy of HGMX to some degree. However, all of these RCTs had a short treatment course (≤ 8 weeks) and were at high risk of selection and performance bias due to a lack of reporting of information regarding allocation concealment, blindness, and data monitoring. Therefore, high-quality RCT evidence is warranted regarding the efficacy of HGMX for patients who have undergone radical gastrectomy for gastric cancer.

This RCT aims to clarify whether HGMX, as a dietary formula based on TCM theory, is effective and safe for improving SQD syndrome, quality of life, performance status, and prognosis of patients with gastric cancer who have undergone radical gastrectomy. To improve the quality of study reporting and conduct, we will develop a standard operating procedure manual according to the principles of the Consolidated Standards of Reporting Trials Extension for Chinese Herbal Medicine Formulas [29] and will thoroughly train all investigators. A potential limitation of this trial is that we will use a modified version of the classic SQD scale that has not been validated (no reliability or validity evaluation).

Based on our pilot observations, many patients undergoing gastrectomy achieved quick remission of their gastrointestinal symptoms with 8 weeks of HGMX treatment. Thus, we decided to use three visits (at weeks 2, 4, and 8) to validate the short-term effects of HGMX, which will also provide data for the early discontinuation of the trial as determined by the DMC. The aim of the visits on weeks 26, 52, 78, and 104 will be to investigate the long-term efficacy and safety of HGMX. To improve long-term patient compliance—the key point for obtaining precise results in this trial—we will only include patients with an ECOG score ≤ 1, which ensures that they have sufficient physical abilities to complete this long-term trial and their gastrointestinal symptoms are mild and tolerable so that the need for additional medicine is infrequent. If this trial proves the efficacy of HGMX in patients with good physical performance, we will consider conducting further trials that focus on patients who have worse physical performance (e.g., ECOG score ≥ 2). Moreover, based on our experience, most patients with gastric cancer will return to the hospital to receive regular postoperative follow-up with their attending physicians. Therefore, we anticipate that good compliance with the protocol can be ensured for most patients during the 2-year follow-up period.

This RCT is one of a series of RCTs (the other two trials will focus on nonorganic gastrointestinal disorders and peptic ulcer diseases) assessing the capacity of HGMX to improve gastrointestinal function and systemic symptoms. Although dietary TCM formulas are widely used in clinical practice in China, RCTs with a rigorous design and long-term follow-up are still lacking. To the best of our knowledge, this RCT will be the first to assess a dietary TCM formula for the management of gastric cancer. The results of the RCTs will provide experience and evidence for developing guidelines and policies for facilitating the reasonable use of dietary TCM formulas.

### Trial status

The protocol version number is 3.2. Trial recruitment was started on May 22, 2017, and the trial has enrolled 80 patients at the time of manuscript submission. Recruitment is expected to be completed in October 2019.

## Additional files

Additional file 1: Spleen Qi Deficiency Symptoms Grading and Quantifying scale. (PDF 221 kb)

Additional file 2: SPIRIT 2013 checklist: recommended items to address in a clinical trial protocol and related documents. (DOCX 51 kb)

## Data Availability

Not applicable.

## Abbreviations

AE: Adverse event
DMC: Data monitoring committee
ECOG: Eastern Cooperative Oncology Group
HGMX: Hou Gu Mi Xi
RCT: Randomized controlled trial
SAE: Severe adverse event
SPIRIT: Standard Protocol Items: Recommendations for Interventional Trials
SQD scale: Spleen Qi Deficiency Symptoms Grading and Quantifying scale
SQD: Spleen qi deficiency
TCM: Traditional Chinese medicine
ULN: Upper limit of normal
