# Supplementary material for: Efficacy and safety of Hou Gu Mi Xi in patients with spleen qi deficiency syndrome who underwent radical gastrectomy for gastric cancer: protocol for a multicenter, randomized, double-blind, placebo-controlled trial
Source: Trials. 2019 Jun 10;20:343. doi: 10.1186/s13063-019-3429-x (PMC6558912; doi:10.1186/s13063-019-3429-x)
Supplement: Supplementary file 1 — Spleen Qi Deficiency Symptoms Grading and Quantifying scale. (PDF 221 kb) [file 13063_2019_3429_MOESM1_ESM.pdf]

Spleen Qi Deficiency Symptoms Grading and Quantifying Scale

Item	Option
**1 Stomach distension***	□$_0$ None
Duration	□$_1$ < 0.5 hour □$_2$ 0.5-1 hour □$_3$ > 1 hour
Severity	□$_1$ Mild □$_2$ Moderate □$_3$ Severe
Frequency per day	□$_1$ Occasionally □$_2$ Sometimes □$_3$ Most of the time □$_4$ Persistently
Frequency per week	□$_1$ < 1 day □$_2$ 1 day □$_3$ 2-3 days □$_4$ 4-5 days □$_5$ 6-7 days
**2 Abdominal distension***	□$_0$ None
Duration	□$_1$ < 0.5 hour □$_2$ 0.5-1 hour □$_3$ > 1 hour
Severity	□$_1$ Mild □$_2$ Moderate □$_3$ Severe
Frequency per day	□$_1$ Occasionally □$_2$ Sometimes □$_3$ Most of the time □$_4$ Persistently
Frequency per week	□$_1$ < 1 day □$_2$ 1 day □$_3$ 2-3 days □$_4$ 4-5 days □$_5$ 6-7 days
**3 Physical Fatigue and weakness***	□$_0$ None
Severity	□$_1$ Mild □$_2$ Moderate □$_3$ Severe
Frequency per day	□$_1$ Occasionally □$_2$ Sometimes □$_3$ Most of the time □$_4$ Persistently
Frequency per week	□$_1$ < 1 day □$_2$ 1 day □$_3$ 2-3 days □$_4$ 4-5 days □$_5$ 6-7 days
**4 Mental fatigue and taciturnity***	□$_0$ None
Severity	□$_1$ Mild □$_2$ Moderate □$_3$ Severe
Frequency per day	□$_1$ Occasionally □$_2$ Sometimes □$_3$ Most of the time □$_4$ Persistently
Frequency per week	□$_1$ < 1 day □$_2$ 1 day □$_3$ 2-3 days □$_4$ 4-5 days □$_5$ 6-7 days
**5 Loss of appetite***	□$_0$ None
Severity	□$_1$ Mild □$_2$ Moderate □$_3$ Severe
Frequency per day	□$_1$ 1 meal □$_2$ 2 meals □$_3$ 3 meals
Frequency per week	□$_1$ < 1 day □$_2$ 1 day □$_3$ 2-3 days □$_4$ 4-5 days □$_5$ 6-7 days
**6 Abnormal stools***	□$_0$ None
Severity	□$_1$ Unshapen stool □$_2$ Loose stool □$_3$ Diarrhea
Frequency per day	□$_1$ 1 time □$_2$ 2 times □$_3$ 3 times □$_4$ ≥ 4 times
Frequency per week	□$_1$ < 1 day □$_2$ 1 day □$_3$ 2-3 days □$_4$ 4-5 days □$_5$ 6-7 days
**7 Stomach pain**	□$_0$ None
Duration	□$_1$ < 0.5 hour □$_2$ 0.5-1 hour □$_3$ > 1 hour
Severity	□$_1$ Mild □$_2$ Moderate □$_3$ Severe
Frequency per day	□$_1$ Occasionally □$_2$ Sometimes □$_3$ Most of the time □$_4$ Persistently
Frequency per week	□$_1$ < 1 day □$_2$ 1 day □$_3$ 2-3 days □$_4$ 4-5 days □$_5$ 6-7 days

(*Continuous*)

Item	Option
**8 Stomach tightness**	☐$_0$ None
Duration	☐$_1$ < 0.5 hour ☐$_2$ 0.5-1 hour ☐$_3$ > 1 hour
Severity	☐$_1$ Mild ☐$_2$ Moderate ☐$_3$ Severe
Frequency per day	☐$_1$ Occasionally ☐$_2$ Sometimes ☐$_3$ Most of the time ☐$_4$ Persistently
Frequency per week	☐$_1$ < 1 day ☐$_2$ 1 day ☐$_3$ 2-3 days ☐$_4$ 4-5 days ☐$_5$ 6-7 days
**9 Abdominal pain**	☐$_0$ None
Duration	☐$_1$ < 0.5 hour ☐$_2$ 0.5-1 hour ☐$_3$ > 1 hour
Severity	☐$_1$ Mild ☐$_2$ Moderate ☐$_3$ Severe
Frequency per day	☐$_1$ Occasionally ☐$_2$ Sometimes ☐$_3$ Most of the time ☐$_4$ Persistently
Frequency per week	☐$_1$ < 1 day ☐$_2$ 1 day ☐$_3$ 2-3 days ☐$_4$ 4-5 days ☐$_5$ 6-7 days
**10 Acid reflux**	☐$_0$ None
Severity	☐$_1$ Mild ☐$_2$ Moderate ☐$_3$ Severe
Frequency per day	☐$_1$ Occasionally ☐$_2$ Sometimes ☐$_3$ Most of the time ☐$_4$ Persistently
Frequency per week	☐$_1$ < 1 day ☐$_2$ 1 day ☐$_3$ 2-3 days ☐$_4$ 4-5 days ☐$_5$ 6-7 days
**11 Belching**	☐$_0$ None
Severity	☐$_1$ Mild ☐$_2$ Moderate ☐$_3$ Severe
Frequency per day	☐$_1$ Occasionally ☐$_2$ Sometimes ☐$_3$ Most of the time ☐$_4$ Persistently
Frequency per week	☐$_1$ < 1 day ☐$_2$ 1 day ☐$_3$ 2-3 days ☐$_4$ 4-5 days ☐$_5$ 6-7 days
**12 Nausea and vomiting**	☐$_0$ None
Severity	☐$_1$ Mild ☐$_2$ Moderate ☐$_3$ Severe
Frequency per day	☐$_1$ Occasionally ☐$_2$ Sometimes ☐$_3$ Most of the time ☐$_4$ Persistently
Frequency per week	☐$_1$ < 1 day ☐$_2$ 1 day ☐$_3$ 2-3 days ☐$_4$ 4-5 days ☐$_5$ 6-7 days
**13 Abnormal bowel sounds**	☐$_0$ None
Frequency per day	☐$_1$ Occasionally ☐$_2$ Sometimes ☐$_3$ Most of the time ☐$_4$ Persistently
Frequency per week	☐$_1$ < 1 day ☐$_2$ 1 day ☐$_3$ 2-3 days ☐$_4$ 4-5 days ☐$_5$ 6-7 days
**14 Powerless defecation**	☐$_0$ None
Severity	☐$_1$ Mild ☐$_2$ Moderate ☐$_3$ Severe
Frequency per day	☐$_1$ Occasionally ☐$_2$ Sometimes ☐$_3$ Most of the time ☐$_4$ Persistently
Frequency per week	☐$_1$ < 1 day ☐$_2$ 1 day ☐$_3$ 2-3 days ☐$_4$ 4-5 days ☐$_5$ 6-7 days
**15 Sallow complexion**	☐$_0$ None
Severity	☐$_1$ Mild ☐$_2$ Moderate ☐$_3$ Severe
Frequency per day	☐$_1$ Occasionally ☐$_2$ Sometimes ☐$_3$ Most of the time ☐$_4$ Persistently
Frequency per week	☐$_1$ < 1 day ☐$_2$ 1 day ☐$_3$ 2-3 days ☐$_4$ 4-5 days ☐$_5$ 6-7 days

(*Continuous*)

Item	Option				
**16 Loss of taste and hypodipsia**	□$_0$ None				
Severity	□$_1$ Mild	□$_2$ Moderate	□$_3$ Severe		
Frequency per day	□$_1$ Occasionally	□$_2$ Sometimes	□$_3$ Most of the time	□$_4$ Persistently	
Frequency per week	□$_1$ < 1 day	□$_2$ 1 day	□$_3$ 2-3 days	□$_4$ 4-5 days	□$_5$ 6-7 days
**17 Facial and limb oedema**	□$_0$ None				
Severity	□$_1$ Mild	□$_2$ Moderate	□$_3$ Severe		
Frequency per day	□$_1$ Occasionally	□$_2$ Sometimes	□$_3$ Most of the time	□$_4$ Persistently	
Frequency per week	□$_1$ < 1 day	□$_2$ 1 day	□$_3$ 2-3 days	□$_4$ 4-5 days	□$_5$ 6-7 days

*: primary symptoms
Reference for assessing severity of symptoms or signs:
Mild: The symptoms or signs are not obvious and patients cannot felt without reminding;
Moderate: The symptoms or signs are obvious but do not affect patients' living and work;
Severity: The symptoms or signs are very obvious and affect patients' living and work.
